# Systematic Review and Meta-Analysis of a Proprietary Alpha-Amylase Inhibitor from White Bean (*Phaseolus vulgaris* L.) on Weight and Fat Loss in Humans

**DOI:** 10.3390/foods7040063

**Published:** 2018-04-20

**Authors:** Jay Udani, Ollie Tan, Jhanna Molina

**Affiliations:** Medical Private Practice, Agoura Hills, CA 91301, USA; ollietan124@yahoo.com (O.T.); jhannamolina@gmail.com (J.M.)

**Keywords:** weight loss, fat loss, obesity, alpha-amylase inhibitor, meta-analysis, *Phaseolus vulgaris* L.

## Abstract

The aim of this meta-analysis was to examine the evidence for the effectiveness of a proprietary alpha-amylase inhibitor from white bean (*Phaseolus vulgaris* L.) supplementation interventions in humans on modification of body weight and fat mass. A systematic literature search was performed using three databases: PubMed, the Cochrane collaboration, and Google Scholar. In addition, the manufacturer was contacted for internal unpublished data, and finally, the reference section of relevant original research and review papers were mined for additional studies. Eleven studies were selected for the meta-analysis of weight loss (a total of 573 subjects), and three studies for the meta-analysis of body fat reduction (a total of 110 subjects), as they fulfilled the inclusion criteria. *Phaseolus vulgaris* supplementation showed an average effect on weight loss difference of −1.08 kg (95% CI (confidence interval), −0.42 kg to −1.16 kg, *p* < 0.00001), and the average effect on body fat reduction was 3.26 kg (95% CI, −2.35 kg to −4.163 kg, *p* = 0.02). This meta-analysis found statistically significant effects of *Phaseolus vulgaris* supplementation on body weight and body fat.

## 1. Introduction

There are many dietary interventions available to counteract the epidemic of overweight and obesity which play a major role in the development of insulin resistance and type 2 diabetes mellitus [1]. One strategy is based on lowering the excessive intake of carbohydrates, especially, the refined ones [2]. This could be achieved by lowering the portions or replacing the carbohydrates with more fats or by adding soluble fiber to the diet which is thought to slow down the absorption of carbohydrates [3]. Lowering the glycemic index through the usage of fiber in the diet is not favored by most people due to potential taste preferences and adverse reactions resulting in gastrointestinal problems such as gas and diarrhea. Therefore, another strategy becomes more and more promising to impact the carbohydrate absorption by using bioactive ingredients which block or slow the carbohydrate absorption in the gastrointestinal tract via inhibiting the necessary enzymes, amylase and glucosidase [4]. Amylase breaks down complex carbohydrates, such as starch, into oligosaccharides and glucosidase enzymes further convert these to monosaccharides.

There are the different forms of amylase inhibitors, namely, Alpha-amylase inhibitor isoform 1 (Alpha-AI1), Alpha-AI2, and Alpha-AIL which can be found in in the embryonic axes and cotyledons in the seed of common beans (*Phaseolus* spp.) [5]. These so-called glycoproteins bind to alpha-amylase non-covalently, mainly through hydrophobic interaction, by completely blocking access to the active site of the alpha-amylase [6,7]. The Alpha-AI1 isoform is the one with anti-amylase bioactivity in humans, and therefore, inhibits the starch digestion [8]. This blocking affect is also dependent on pH, temperature, incubation time and the presence of particular ions which have been optimized for the specific and proprietary product named Phase2^®^ brand *Phaseolus vulgaris* White Bean product (Pharmachem Laboratories, Kearny, NJ, USA) [9,10]. This particular dietary supplement has demonstrated its potential and ability to cause weight loss in numerous clinical trials in humans [11].

Phase2^®^ brand *Phaseolus vulgaris* White Bean extract is made by a standardized water extract of non-GMO (Genetically Modified Organism) whole dried beans (*Phaseolus vulgaris*) which is made through a proprietary process. The white-to-beige powder consists of *Phaseolus vulgaris* (~90%) and Gum Arabic (~10%). It has at least 3000 alpha-amylase inhibiting units (AAIU) per gram when tested at a pH 6.8 using potato starch as the substrate and pancreatin as the enzyme source. The Phase2^®^ brand products are used in dietary supplements in various forms, including powders, tablets, capsules and chewables for the application of weight control and weight loss. In addition, it is also incorporated in food products like chewing gum, mashed potatoes, yeast-raised dough (bread, pizza, etc.) without losing bioactivity or changing the appearance, texture or taste of the food [12,13,14].

The aim of this meta-analysis was to examine the evidence for the effectiveness of a proprietary alpha-amylase inhibitor from white bean (*Phaseolus vulgaris*) supplementation interventions on modification of body weight and fat mass.

## 2. Methods

This review was performed according to the PRISMA (preferred reporting items for systematic reviews and meta-analyses) statement for quality of reporting a meta-analysis [15].

### 2.1. Literature Search

Literature searches in PubMed, the Cochrane collaboration, and Google Scholar were undertaken using the following keywords: *Phaseolus vulgaris*, Alpha-amylase inhibitor/inhibition, Phase2^®^, White bean extract, kidney bean, starch blocker, weight loss, body weight, body fat, BMI (body mass index), anthropometric measures, obesity, overweight and safety.

In addition, the manufacturer was contacted for internal unpublished data, and finally, the reference section of relevant original research and review papers were mined for additional studies. No age, sex, geographic, time or publication status restrictions were imposed on the initial search. 

### 2.2. Study Selection Criteria

Studies were eligible for inclusion if they met the following PICOS’ (Participants, Intervention, Control, Outcome measurements, and Study design) criteria: (a) Participants: overweight or obese individuals; (b) Intervention: Phase2^®^ brand *Phaseolus vulgaris* white bean extract, at least 1200 mg per day, for at least 4 weeks; (c) Control: studies comparing the experimental group (*Phaseolus vulgaris* supplementation) with a control/placebo group (no *Phaseolus vulgaris* supplementation ), or against baseline; (d) Outcome measurements: studies needed to include measurements of body mass or fat mass; (e) Study design: Studies needed to be either randomized, double-blind, placebo-controlled parallel or crossover trial, or open-label studies.

### 2.3. Assessment of Risk of Bias

For the quality assessment of randomized controlled trials (RCTs), we used the Delphi list, which includes eight questions with three response options “yes”, “no”, or “do not know” depending on compliance with key methodological components, and produces a quality score of maximum 9 points that provides an overall estimate of RCT quality [16]. 

### 2.4. Data Extraction and Quality Assessment

Two reviewers independently extracted the following data from the selected articles: publication year, number of participants (*Phaseolus vulgaris* and control group), baseline characteristics of the participants, methodological characteristics of the study, pre- and post-values and standard deviation for body mass and fat mass, and statistical information.

### 2.5. Statistical Analysis

A meta-analysis to estimate the overall treatment effect of *Phaseolus vulgaris* supplementation relative to control groups was performed. Standardized Mean Difference (d) was used in the determination of effect size. In getting the Standardized Mean Difference (d), Cramer’s *v* (which shows the magnitude size) and 95% CI (confidence interval) for each d was computed. The following guideline was used in reading magnitude effect size for Cramer’s *v*: *v* < 0.1 small effect, 0.1 < *v* < 0.3 medium effect, *v* > 0.3 large effect. Weighted effect size on all studies was done using the Hunter–Schmidt approach. Weights are objectively assigned based from sample sizes of the studies. Hence, studies with bigger sample size (*n* = 60 [17]) had higher weight, compared to studies with smaller sample size (*n* = 10 [18]). *p*-values of individual studies are transformed (logarithmic) and aggregated using Chi-square.

## 3. Results

### 3.1. Article Selection

**Figure 1:**
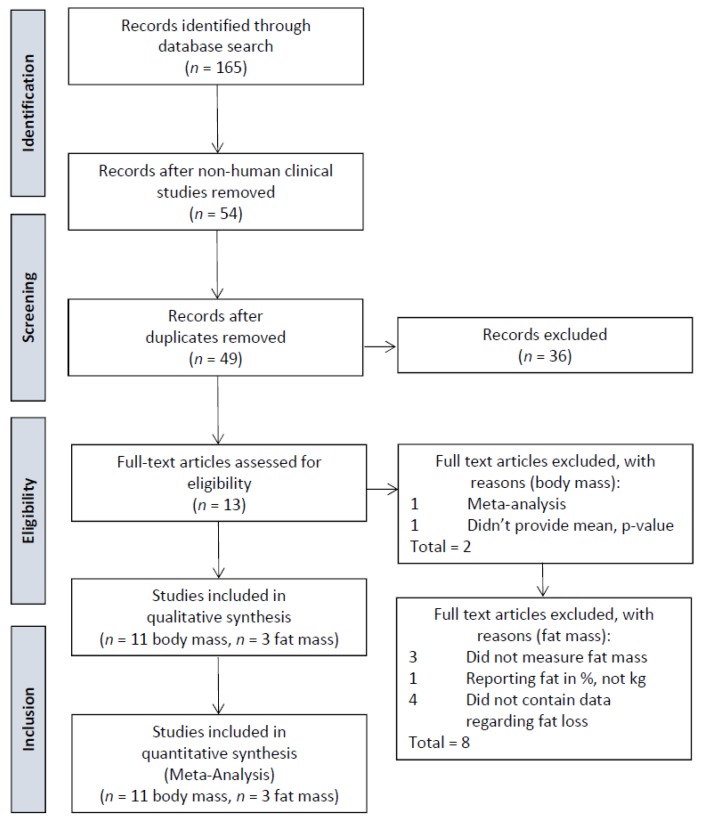
Prisma flow diagram for *Phaseolus vulgaris* and body mass, fat mass.

One hundred and sixty-five articles were identified. From this list, 54 human studies were identified, and 5 of these were determined to be duplicates, leaving 49 unique studies. Of these 49 studies, only 13 involved the Phase2^®^ *Phaseolus vulgaris* ingredient and one of the following outcomes: weight loss, body fat loss, or anthropometric measures (reductions in waist, hip or thigh measurements). A meta-analysis which included studies which did and did not use the Phase2^®^ *Phaseolus vulgaris* ingredient was excluded. In addition, one of the studies was excluded as it did not provide means, standard deviations, nor *p*-values [19]. The meta-analysis for weight loss includes 11 studies (see prisma flow diagram, Figure 1), with a total of 573 subjects (see Table 1). The meta-analysis for fat loss includes 3 studies [18,20,21] with a total of 110 subjects (see Table 2). Three studies were excluded, as they did not measure fat mass [22,23,24]. One study was excluded as it only reported fat loss in percent and not in kilogram [25]. 

### 3.2. Phaseolus vulgaris Doses and Duration of Supplementation

The most common dose of *Phaseolus vulgaris* was 3000 mg per day, divided into three doses of 1000 mg (6/11). One study used 3000 mg per day, divided into two doses of 1500 mg [18], and one study used 2000 mg per day, divided into two doses of 1000 mg [11]. Two studies used 400 mg [21], and 445 mg [20] *Phaseolus vulgaris*, respectively, as part of a multi-ingredient blend. One study did not specify the amount of *Phaseolus vulgaris* used [25]. *Phaseolus vulgaris* was supplemented for 1 month (3/11) [11,20,23], 2 months (5/11) [18,24,25,26,28], or 3 months (3/11) (see Table 3) [17,21,27].

### 3.3. Control Groups

Participants of the control group had similar characteristics to the intervention groups, but they did not receive *Phaseolus vulgaris* supplementation. In most studies, placebo capsules were administered.

### 3.4. Effects on Body Weight

Table 1 summarizes the effects of *Phaseolus vulgaris* on body mass. *Phaseolus vulgaris* supplementation showed an average effect on weight loss difference of −1.08 kg (95% CI, −0.42 kg to −1.16 kg, *p* < 0.00001).

### 3.5. Effects on Fat Loss

Table 2 summarizes the effects of *Phaseolus vulgaris* on fat mass. The average effect of *Phaseolus vulgaris* supplementation on body fat reduction was 3.26 kg (95% CI, −2.35 kg to −4.163 kg, *p* = 0.02).

### 3.6. Risk of Bias and Publication Bias

Table 4 shows Delphi scores of each reviewed study. The Delphi scores varied between 1 and 9, the mean being 5.9 and the standard deviation ±3.2. Four studies obtained a score below the mean: Asano [26], Koike et al. 2005 [18], Osorio et al. 2009 [23], and Yamada et al. [25], with Asano [26], Koike et al. [18] and Osorio et al. [23] being open-label studies.

## 4. Discussion

The aim of this meta-analysis was to determine the effectiveness of Phase2^®^ (*Phaseolus vulgaris*) to support weight loss and to reduce body fat. The overall meta-analysis revealed a significant difference in change in body weight, and body fat between Phase2^®^ (*Phaseolus vulgaris*) and placebo.

Barret et al. conducted a review of clinical studies with Phase 2 brand *Phaseolus vulgaris* White Bean product on weight loss and glycemic control [29]. The analysis identified ten clinical studies which have demonstrated weight loss over time following administration of Phase 2 when taken concurrently with meals containing carbohydrates. Three of these clinical studies revealed significant loss of body weight with Phase 2 compared to a placebo control in people who are overweight or obese. In addition, three clinical trials showed a reduction in serum triglycerides over time. Nine of these clinical studies reported by Barret et al. have been used in this systematic review and meta-analysis. The study by Vinson et al. was not taken into consideration due to its focus on glycemic index and blood glucose investigations without looking into weight loss parameters [30].

While our meta-analysis revealed a significant difference in weight loss over placebo, a previous meta-analysis of *Phaseolus vulgaris* [31] showed a non-significant difference in weight loss between *Phaseolus vulgaris* and placebo groups. This can be explained by the fact that the Onakpoya et al. meta-analysis included not only studies performed with Phase 2, but all studies on *Phaseolus vulgaris*. Both meta-analyses showed significant effects on fat loss. The importance of this work was to isolate the effects of the Phase 2 brand *Phaseolus vulgaris* White Bean from the body of literature. By using unpublished data and all arms of all studies available to us, we were able to demonstrate statistically significant effects on weight and body fat. Part of this importance is in the supplement industry, there is an assumed “generic equivalence” which we know to be false. The prior meta-analysis assumed such a generic equivalence and therefore came up with negative results. In this case, by limiting to only Phase 2, it does appear that there is significant weight and body fat loss with an excellent safety profile.

Low carbohydrate diets have been linked to weight loss, even when not consciously restricting calories, improved triglyceride levels, a reduction in blood glucose levels and improved insulin sensitivity, a decrease in blood pressure. Very low carbohydrate diets (ketogenic diets), with fewer than 50 g of carbohydrate per day, have been linked to weight loss and specific health benefits including neurological disorders. Adaptations to a ketogenic diet is often difficult and nutritional aids have been shown to be useful for entering into nutritional ketosis [32]. A recently concluded study indicated that both low-fat and low-carb diets can work for weight loss, and that there is no “best diet” when it comes to low-carb vs. low-fat diets. In total, 263 males and 346 premenopausal females were assigned to either a low-fat diet or a low-carb diet for 12 months. At 12 months, the low-fat group had lost 5.3 kg and the low-carb group 6.0 kg and this difference is neither statistically significant nor clinically relevant. The healthy diet that will work for you is the one you can stick to, and that varies by individual [33].

## 5. Conclusions

This meta-analysis found significant effect of Phase2^®^ *Phaseolus vulgaris* supplementation on body weight and body fat.

## Figures and Tables

**Table 1:** Effects of *Phaseolus vulgaris* on body weight. The overall *p*-value was determined using Chi-square (Chi-square value (*W*) = 80.02).

Study	Treatment Group			Control Group			*p*	Weight	Effect (d)	Weighted Mean Difference (Fixed) 95% CI	
	*n*	Mean	SD	*n*	Mean	SD				Lower	Upper
Udani et al. 2007 [11]	13	−6.0		12	−4.7		0.424	4%	−0.33	−0.46	1.12
Asano [26]	9	−2.9						3%	−0.19	−1.06	0.67
Koike et al. 2005 [18]	10	−1.8					0.002	3%	−1.61	−0.61	−2.62
Grube et al. 2014 [17]	60	−2.9	2.6	60	−0.9	2.0	0.001	19%	−0.85	−0.48	−1.12
Osorio et al. 2009 [23]	49	−2.3		49	2.21		0.001	15%	−1.00	−0.41	−1.60
Rothacker (week 12) 2003 [27]	30	−6.9		60	0.8		0.029	9%	−0.58	−0.62	−1.09
Wu et al. 2010 [24]	50	−1.9	−0.2	51	−0.4	−0.1	0.049	15%	−0.40	-0.00	−0.79
Celleno et al. 2007 [20]	20	−2.9	−1.2	30	−0.4	0.4		9%	−2.99	−2.25	−3.73
Thom et al. 2000 [21]	20	−3.5		20	2.0		0.001	6%	−1.13	−0.46	−0.12
Udani et al. 2004 [28]	20	−3.8		19	−1.65		0.35	6%	−0.30	−0.33	−0.93
Yamada et al. [25]	33	−0.8	0.2	33			0.01	10%	−0.97	−0.24	−1.68
Total	314			259			0.001	100%	−1.08	−0.43	−1.16

SD: standard deviation, CI: confidence interval.

**Table 2:** Effects of *Phaseolus vulgaris* on body fat. The overall *p*-value was determined using Chi-square (Chi-square value (*W*) = 36.84).

Study	Treatment Group			Control Group			*p*	Weight	Effect (d)	Weighted Mean Difference (Fixed) 95% CI	
	*n*	Mean	SD	*n*	Mean	SD				Lower	Upper
Koike et al. 2005 [18]	10	−1.2	−0.4				0.001	17%	−1.58	−2.58	−0.57
Celleno et al. 2007 [20]	30	−2.4	−0.67	30	−0.16	−0.33	0.001	50%	−4.24	−5.15	−3.33
Thom et al. 2000 [21]	20	−2.3	−1.5	20	0.7	−0.6	0.01	33%	−2.63	−3.47	−1.78
Total	60			50			0.02	100%	−3.26	−4.16	−2.35

**Table 3:** Characteristics of the 11 clinical studies included in the meta-analysis.

Study	Participants		Intervention				Comparison	Methods		Study Design	
	Country	Subjects Information	Dose	Diet Intervention	Duration of Intervention	*n* Phase 2	*n* Control	Weight	Fat Mass	Design	Delphi-Score
Asano et al. [26]	Japan	5:1 female to male ratio; average age 36.3 + 12.7; BMI > 25; average BMI = 31.6	3000 mg per day (1000 mg per meal)	no caloric restriction	2 months	9	0	Scale	n/a	Open-Label	2
Udani et al. 2007 [22]	USA	0.3:1 female to male ratio; age 18-40; average BMI = 26	2000 mg per day (1000 mg at breakfast & lunch)	maintain a caloric intake of 1800 per day	4 weeks	13	12	Scale	-	RDBPC	8
Koike et al. 2005 [18]	Japan	1:1 female to male ratio; mean age 41.1 and BMI range 23–30	2× per day 1500 mg Phase 2, 400 mg Clove, 40 mg Lysine 40 mg, 40 mg Arginine, 40 mg Alanine	no caloric restriction	8 weeks	10	0	Scale	n/a	Open-Label	1
Grube et al. 2014 [17]	Germany	3:1 female to male ratio; mean age 46; BMI range 25–35	3000 mg per day (1000 mg per meal)	hypocaloric (500 kcal), providing 40% of energy as carbohydrates	12 weeks	60	57	Scale	BIA	RDBPC	9
Osorio et al. 2009 [23]	Mexico	obese and overweight (age range 18–75 years)	3000 mg per day (1000 mg per meal)	no caloric restriction besides carbohydrate-rich meals	30 days	37	0	Scale	-	Open-Label	1
Rothacker 2003 [27]	USA	24 male; 36 female; mean age 33.2; BMI range 24–32	3000 mg per day (1000 mg per meal)	no caloric restriction	12 weeks	34	26	Scale	BIA	RDBPC	8
Wu et al. 2010 [24]	China	1:1 female to male ratio; age 20-50; BMI range 25–40	3000 mg per day (1000 mg per meal)	no caloric restriction	8 weeks	51	50	Scale	-	RDBPC	8
Celleno et al. 2007 [20]	Italy	2.5:1 female to male ratio; mean age 34; average BMI = 26	3× per day 445 mg of Phase 2, 56 mg vitamin B3, and 0.5 mg chromium	carbohydrate-rich meals (100–200g)	30 days	30	29	Scale	BIA	RDBPC	8
Thom et al. 2000 [21]	Norway	9:1 female to male ratio; mean age 45.6; average BMI = 31	3× per day 400 mg Phase 2, 400 mg inulin, and 100 mg Garcinia cambogia	no caloric restriction	12 weeks	20	20	Scale	BIA	RDBPC	8
Udani et al. 2004 [28]	USA	9:1 female to male ratio; mean age 36.5; average weight of 193.1 pounds	3000 mg per day (1000 mg per meal)	no caloric restriction	8 weeks	20	19	Scale	BIA	RDBPC	8
Yamada et al. [25]	Japan	1:1 female to male ratio; age 25–60; no BMI information	Twice a day proprietary functional food containing Phase 2	no caloric restriction	8 weeks	23	24	Scale	n/a	Open-Label	4

BMI: body mass index; RDBPC: Randomized, Double-Blind, Placebo-Controlled; n/a: method not described; BIA: bioelectrical impedance analysis; -: not measured.

**Table 4:** Delphi-Scores.

Delphi-Scores/Studies	Asano et al. [26]	Udani et al. 2007 [11]	Grube et al. 2014 [17]	Rothacker et al. 2003 [27]	Wu et al. 2010 [24]	Celleno et al. 2007 [20]	Thom et al. 2000 [21]	Udani et al. 2004 [28]	Koike et al. 2005 [18]	Osorio et al. 2009 [23]	Yamada et al. [25]
1. Treatment allocation (a) Was a method of randomization performed?	0	1	1	1	1	1	1	1	0	0	1
(b) Was the treatment allocation concealed?	0	1	1	1	1	1	1	1	0	0	0
2. Were the groups similar at baseline regarding the most important prognostic indicators?	0	1	1	1	1	1	1	1	0	0	1
3. Where the eligibility criteria specified?	1	1	1	1	1	1	1	1	0	0	1
4. Was the outcome assessor blinded?	0	1	1	1	1	1	1	1	0	0	0
5. Was the care provider blinded?	0	1	1	1	1	1	1	1	0	0	0
6. Was the patient blinded?	0	1	1	1	1	1	1	1	0	0	0
7. Were point estimates and measures of variability presented for the primary outcome measures?	1	1	1	1	1	1	1	1	1	1	1
8. Did the analysis include an intention-to-treat analysis?	0	0	1	0	0	0	0	0	0	0	0
Total Delphi Score	2	8	9	8	8	8	8	8	1	1	4

## References

[1] De Toro-Martín J., Arsenault B.J., Després J.P., Vohl M.C. (2017). Precision Nutrition: A Review of Personalized Nutritional Approaches for the Prevention and Management of Metabolic Syndrome. Nutrients.

[2] Preuss H.G., Bagchi D., Bronner F. (2002). Nutritional therapy of impaired glucose tolerance and diabetes mellitus. Nutritional Aspects and Clinical Management of Chronic Disorders and Diseases.

[3] Bell S.J., Sears B. (2003). Low-Glycemic-Load Diets: Impact on Obesity and Chronic Diseases. Crit. Rev. Food Sci. Nutr..

[4] Fukagawa N.K., Anderson J.W., Hageman G., Young V.R., Ninaker K.L. (1990). High-carbohydrate, high-fiber diets increase peripheral insulin sensitivity in healthy young and old adults. Am. J. Clin. Nutr..

[5] Bowman D.E. (1945). Amylase inhibitor of navy bean. Science.

[6] Santimone M., Koukiekolo R., Moreau Y., Le Berre V., Rouge P., Marchis-Mouren G., Desseaux V. (2004). Porcine pancreatic a-amylase inhibition by the kidney bean (*Phaseolus vulgaris*) inhibitor (α-AI1) and structural changes in the α-amylase inhibitor complex. Biochim. Biophys. Acta.

[7] Bompard-Gilles C., Rousseau P., Rouge P., Payan F. (1996). Substrate mimicry in the active center of a mammalian α-amylase: Structural analysis of an enzyme-inhibitor complex. Structure.

[8] Payan F. (2004). Structural basis for the inhibition of mammalian and insect α-amylases by plant protein inhibitors. Biochim. Biophys. Acta.

[9] Le Berre-Anton V., Bompard-Gilles C., Payan F., Rouge P. (1997). Characterization and functional properties of the α-amylase inhibitor (α-AI) from kidney bean (*Phaseolus vulgaris*) seeds. Biochim. Biophys. Acta.

[10] Lajolo F.M., Finardi-Filho F. (1985). Partial characterization of the amylase inhibitor of black beans (*Phaseolus vulgaris*), variety Rico 23. J. Agric. Food Chem..

[11] Udani J., Hardy M., Kavoussi B., Bagchi D., Preuss H.G. (2007). Dietary supplement carbohydrate digestion inhibitors: A review of the literature. Obesity. Epidmiology, Pathophysiology, and Prevention.

[12] Udani K. (2005). The Mighty Bean.

[13] Phase 2^®^/StarchLite. www.phase2info.com/pdf/Phase2_Study13.pdf.

[14] Phase 2^®^/StarchLite in Chewing Gum. http://www.phase2info.com/pdf/Phase2_Study14.pdf.

[15] Moher D., Liberati A., Tetzlaff J., Altman D.G., the PRISMA Group (2009). Preferred Reporting Items for Systematic Reviews and Meta-Analyses: The PRISMA Statement. Ann. Intern. Med..

[16] Verhagen A.P., de Vet H.C.W., de Bie R.A., Kessels A.G., Boers M., Bouter L.M., Knipschild P.G. (1998). The Delphi list: A criteria list for quality assessment of randomized clinical trials for conducting systematic reviews developed by Delphi consensus. J. Clin. Epidemiol..

[17] Grube B., Chong W., Chong P., Riede L. (2014). Weight Reduction and Maintenance with IQP-PV-101: A 12-Week Randomized Controlled Study with a 24-Week Open Label Period. Obesity.

[18] Koike T., Koizumi Y., Tang L., Takahara K., Saitou Y. (2005). The antiobesity effect and the safety of taking “Phaseolamin(TM) 1600 diet”. J. New Rem. Clin..

[19] Erner S., Meiss D. (2003). The Effect of Thera-Slim on Weight, Body Composition and Select Laboratory Parameters in Adults with Overweight and Mild-Moderate Obesity.

[20] Celleno L., Tolaini M.V., D’Amore A., Perricone N.V., Preuss H.G. (2007). A dietary supplement containing standardized Phaseolus vulgaris extract influences body composition of overweight men and women. Int. J. Med. Sci..

[21] Thom E. (2000). A randomized, double-blind, placebo-controlled trial of a new weight-reducing agent of natural origin. J. Int. Med. Res..

[22] Udani J., Singh B. (2007). Blocking Carbohydrate Absorption and Weight Loss: A Clinical Trial Using a Proprietary Fractionated White Bean Extract. Altern. Ther. Health Med..

[23] Osorio L., Gamboa J. (2005). Random Multi-Center Evaluation to Test the Efficacy of *Phaseolus vulgaris* (Precarb) in Obese and Overweight Individuals.

[24] Wu X., Xiaofeng X., Shen J., Perricone N., Preuss H. (2010). Enhanced Weight Loss From a Dietary Supplement Containing Standardized *Phaseolus vulgaris* Extract in Overweight Men and Women. J. Appl. Res..

[25] Yamada J., Yamamoto T., Hideyo Y. (2007). Effects of Combination of Functional Food Materials on Body Weight, Body Fat Percentage, Serum Triglyceride and Blood Glucose.

[26] Asano N. (2009). The Report of the Test Regarding the Efficacy of Phaseolamine on Weight Loss.

[27] Rothacker D. (2003). Reduction in body weight with a starch blocking diet aid: StarchAway comparison with placebo. Leiner Health Prod..

[28] Udani J., Hardy M., Madsen D.C. (2004). Blocking carbohydrate absorption and weight loss: A clinical trial using Phase 2 brand proprietary fractionated white bean extract. Altern. Med. Rev..

[29] Barrett M.L., Udani J.K. (2011). A proprietary alpha-amylase inhibitor from white bean (*Phaseolus vulgaris*): A review of clinical studies on weight loss and glycemic control. Nutr. J..

[30] Vinson J., Al Kharrat H., Shuta D. (2009). Investigation of an amylase inhibitor on human glucose absorption after starch consumption. Open Nutraceuticals J..

[31] Onakpoya I., Aldaas S., Terry R., Ernst E. (2011). The efficacy of Phaseolus vulgaris as a weight-loss supplement: A systematic review and meta-analysis of randomised clinical trials. Br. J. Nutr..

[32] Harvey C.J.D.C., Schofield G.M., Williden M. (2018). The use of nutritional supplements to induce ketosis and reduce symptoms associated with keto-induction: A narrative review. PeerJ.

[33] Gardner C.D., Trepanowski J.F., Del Gobbo L.C., Hauser M.E., Rigdon J., Ioannidis J.P.A., Desai M., King A.C. (2018). Effect of Low-Fat vs Low-Carbohydrate Diet on 12-Month Weight Loss in Overweight Adults and the Association With Genotype Pattern or Insulin Secretion. JAMA.

